# A Retrospective Multicenter Study of Arterial Thromboembolic Events in Hospitalized COVID-19 Patients: Incidence and Imaging Characteristics

**DOI:** 10.1007/s00062-025-01503-w

**Published:** 2025-03-04

**Authors:** David Schinz, Marcel Ploch, Andreas Saleh, Philipp Paprottka, Karl-Ludwig Laugwitz, Tareq Ibrahim, Maria Berndt-Mück, Isabelle Riederer, Michael Uder, Christian Maegerlein, Jan Kirschke, Claus Zimmer, Tobias Boeckh-Behrens

**Affiliations:** 1 Department of Neuroradiology, School of Medicine, Technical University of Munich, Ismaninger Str. 22, 81675 Munich, Germany; 2 Institute of Radiology, University Hospital Erlangen, Friedrich-Alexander-Universität Erlangen-Nürnberg (FAU), Maximiliansplatz 3, 91054 Erlangen, Germany; 3 Institute of Diagnostic and Interventional Radiology and Pediatric Radiology, Munich Clinic Schwabing, Kölner Platz 1, 80804 Munich, Germany; 4 Department of Interventional Radiology, School of Medicine, Technical University of Munich, Ismaninger Str. 22, 81675 München, Germany; 5 Department of Internal Medicine I—Cardiology, School of Medicine, Technical University of Munich, Ismaninger Str. 22, 81675 Munich, Germany

**Keywords:** COVID-19, Arterial thrombosis, Sars-CoV‑2, Embolism, Multicenter

## Abstract

**Objectives:**

Throughout the pandemic, it has become evident that COVID-19 should be recognized as a systemic disease that can affect the coagulation system, potentially resulting in arterial thrombotic events (ATE) with partially bulky free-floating clots. This study aimed to investigate the incidence and imaging characteristics of ATE in hospitalized patients with COVID-19 using clinical and imaging data.

**Methods:**

From January 2020 to May 2021, databases of five German tertiary care centers were retrospectively screened for COVID-19 patients with coincidental ATE. ATE were analyzed for localization, time of occurrence, imaging characteristics, and associations with clinical data and laboratory parameters.

**Results:**

Out of 3267 patients, 110 ATE (102 patients, mean age, 72.01 ± 15.64 years; 63 men) were observed in the presence of COVID-19 (3.1%). ATE included ischemic stroke (40%), myocardial infarction (46.4%, %), peripheral infarction (3.6%), thrombi in precerebral arteries (3.6%), mesenteric ischemia (2.7%), thrombi in the aorta (1.8%), splenic infarction (0.9%), and kidney infarction (0.9%). The median time interval between the onset of typical respiratory COVID-19 symptoms and ATE was four days (range, −5–58, negative values indicate ATE prior to symptom onset). A significant percentage of patients exhibited ATEs with an atypical free-floating appearance (10.0%) and multiple occlusions (21.2%).

**Conclusion:**

COVID-19 is a systemic disease associated with ATE in all vascular regions, with a predilection for the heart and brain. The incidence of ATE might be higher than in comparable viral infections and ATE possibly exhibit distinct imaging features rarely seen, such as bulky free-floating clot masses and multiple occlusions. ATE occur most frequently during the first week around the COVID-19 diagnosis.

**Supplementary Information:**

The online version of this article (10.1007/s00062-025-01503-w) contains supplementary material, which is available to authorized users.

## Abstract

- COVID-19 is a systemic disease that can lead to arterial thrombotic events in all vascular regions, which may have distinct morphological features assessable by radiological imaging.

## Key Results

- COVID-19 can lead to arterial thrombotic events (ATE) in all vascular regions of the body, with a predilection for the heart and brain.
- ATE occur most frequently during the first week of COVID-19 diagnosis.
- Arterial thrombosis in COVID-19 may have distinct morphological features.

## Introduction

Although in common perception, respiratory tract symptoms seem to dominate the course of a coronavirus disease 2019 (COVID-19) infection, it became increasingly evident that the virus causes systemic disease, particularly affecting the coagulation system with notable rates of venous and arterial thrombotic events (ATE).

These thromboembolic complications are often, but not always, associated with a severe COVID-19 infection and affect all vascular subsystems to various degrees: macro- and microcirculation, arterial and venous systems [1–3]. Overall, ATE rates are reported to be around 3% [4, 5]. While previous research has mainly concentrated on the two organ systems considered the most relevant—brain and heart—, contemporary large multicenter studies regard the disease as systemic, affecting all parts of the body even weeks after COVID-19 diagnosis [6, 7]. Additionally, although there are extensive multicenter retrospective studies on the time course of the occurrence of ATE, which indicate an increased risk of ATE even weeks after COVID-19 diagnosis [6, 7], these studies lack more detailed analyses on thrombus type or patient-specific risk factors.

Besides the considerably high incidence of COVID-19-associated ATE, the morphological imaging appearance of the associated clots seems to differ in some cases from known typical presentations of ATE. However, there needs to be a more systematic evaluation of this hypothesis. Current evidence for this phenomenon is based on case reports and small single-center studies, reporting huge thrombi in large and medium-sized vessels, including the aorta, aortic branches, and common/internal carotid arteries, several partially adhesive to the vessel walls and with significant floating parts [8–15].

Using detailed clinical and radiological imaging data from five german centers of tertiary care with a total of over 3267 hospitalized patients with COVID-19, we intend to clarify the overall incidence, the time course, the most affected patient groups and the specific imaging characteristics of whole-body ATE associated to a SARS-CoV‑2 virus infection.

## Material and Methods

This HIPAA-compliant retrospective study was carried out following the Declaration of Helsinki and was approved by the local institutional review boards. The need for written informed consent was waived.

### Study Design and Participants

This study was conducted among all hospitalized COVID-19 patients of five centers of tertiary care in the period from the beginning of the COVID-19 pandemic, January 1, 2020, to May 31, 2021. The group of five centers included three with comprehensive stroke centers (CSCs) covering the majority of hospitalized patients in a big metropolitan area (C1: TUM Universitätsklinikum rechts der Isar München, C2: München Klinik Harlaching, C3: München Klinik Bogenhausen) and two with dedicated centers of infectious diseases (CIDs) (C4: München Klinik Schwabing, C5: München Klinik Neuperlach).

**Fig. 1:**
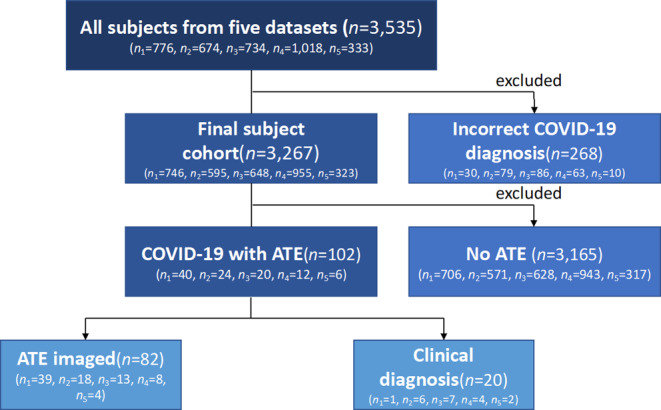
Flow diagram of the investigated study cohort. The Figure shows the flow diagram for subject inclusion and exclusion from top to bottom. Patients were included from five centers of tertiary care (*n*_1–5_) with a total of 3267 subjects (*n*), as 268 subjects had to be excluded because of an incorrect COVID-19 diagnosis

Overall, 3535 hospitalized patients from the five centers (C1: *n* = 776 patients, C2: *n* = 674, C3: *n* = 734, C4: *n* = 1018, C5: *n* = 333) were enrolled. Datasets were distributed by all centers and analyzed centrally. When reviewing the electronic patient records, 268 of the 3535 patients had to be eliminated due to incorrect COVID-19 diagnosis. This resulted in a total number of 3267 patients (C1: *n* = 746, C2: *n* = 595, C3: *n* = 648, C4: *n* = 955, C5: *n* = 323) (see Fig. 1).

The inclusion criteria were a confirmed SARS-CoV‑2 infection by polymerase chain reaction test (PCR). Furthermore, all patients with co-occurring ATE between seven days before the first COVID-19-like symptoms or COVID-19 PCR were included as defined by the upper limit of the confidence interval for the incubation period [16]. A seven-day period before the first COVID-19-like symptoms or COVID-19 positive PCR testing was used to include patients that already developed hypercoagulability, ensuring that pre-symptomatic thrombotic events were not overlooked. Patients with an ATE more than seven days before the first positive test were excluded to minimize the likelihood of unrelated thrombotic events. This resulted in a final cohort of 102 patients with an ATE in the presence of a COVID-19 infection.

### Clinical Variables

Routine healthcare data were collected from electronic medical records. Specifically, clinical variables obtained from the patients’ personal history included baseline characteristics (age, sex), comorbidities, medication, potential prothrombotic risk factors (sepsis, disseminated intravascular coagulation, active cancer, chronic renal failure, prothrombotic drugs, mechanical ventilation and stay at intensive care unit (ICU)), the type and time course of thrombotic events, the dose and type of anticoagulation used before and after ATE, the kind of treatment after ATE, the presence of typical COVID-19 symptoms (cough, fever, loss of smell or taste, dyspnea), the presence of a SARS-CoV 2 related pneumonia, the hospital discharge or death, the interval between first symptoms of COVID-19 infection and ATE, and laboratory parameters (earliest one day before and latest one day after the day of ATE).

### Imaging Data

All available imaging data of ATE, including CT Angiography, Digital Subtraction Angiography (DSA), or MR Angiography, were evaluated. Occlusion locations and number of occlusions per patient were documented, as well as the size and length of vessel occluding clots, degree of wall adherence, and the occurrence of free-floating clot parts with surrounding contrast media. Distinctive morphological features of the thrombi were independently assessed by two neuroradiologists (TBB and DS) with 20 and 4 years of clinical experience in diagnosing vascular disease and arterial embolism. Imaging was performed locally in all five centers.

## Results

### Sample Characteristics

Group demographics and clinical characteristics are presented in Table 1. A table with different group characteristics according to center type (CSC vs. CID) and more in-depth description of anticoagulation medication is provided as Supplementary Table 1.

**Table 1:** Demographical and clinical data of the study cohort

	Hospitalized COVID-19 patients with ATE *n* *=* *102 (%)*
Age (mean ± SD)	72.01 ± 15.64
Sex (f/m)	39/63
*Cardiovascular risk factors*	85 (83.3)
Arterial hypertension	57 (55.9)
Diabetes mellitus	65 (63.7)
Dyslipidemia	11 (10.8)
Smoking	11 (10.8)
Hypercholesterolemia	25 (24.5)
Adiposities	15 (14.7)
Atrial fibrillation	19 (18.6)
Chronic Heart Disease	29 (28.4)
*Treatment at admission*	
None	42 (41.2)
Antiplatelet	42 (41.2)
Oral anticoagulation	14 (13.7)
Statin	56 (54.9)
*Prothrombotic risk factors*	
Active cancer	9 (8.8)
Sepsis	2 (2)
Chronic renal failure	15 (14.7)
MV prior to ATE	9 (8.8)
Duration of MV prior to ATE (mean ± SD) *n* *=* *9*	13.01 ± 14.95
Prothrombotic drugs prior to ATE	18 (17.6)
ICU admission prior to ATE	11 (10.8)
ICU duration prior ATE (mean ± SD) *n* *=* *12*	12.39 ± 15.7
*COVID-19 severity*	
Respiratory symptoms	81 (79.4)
Radiological lung manifestations	83 (81.4)
In-hospital mortality	35 (34.3)

Abbreviations: *ASA* acetylsalicylic acid; *ATE* arterial thromboembolic event; *CID* center of infectious diseases; *COVID-19* coronavirus disease 2019; *CSC* comprehensive stroke centers; *DIC* disseminated intravascular coagulation; *f* female; *ICU* intensive care unit; *m* male; *mg* milligram; *MV* mechanical ventilation; *SD* standard deviation

In brief, the mean age and standard deviation of the study cohort was 72.01 ± 15.56 years (median 76, range 11–102). Included were 39 female and 63 male patients. The most frequent comorbidities included diabetes mellitus (63.7%) and arterial hypertension (55.9%).

At the end of data collection, 34.3% (*n* = 35) of patients with ATE had died. Throughout the entire hospitalization, 40 (39.2%) patients required ICU admission with mechanical ventilation, 36 (35.3%) required ICU admission without mechanical ventilation, and 26 (25.5%) did not require ICU admission.

At the time of ATE, 55 of 100 (55%) patients were under ongoing pharmacological thromboprophylaxis (Table 2). The most frequently received thromboprophylaxis regimen was Acetylsalicylic Acid (100 mg once daily; 33.3%).

**Table 2:** Morphological data of imaged arterial thrombosis

Morphology of arterial thrombosis (60 thrombi in 52 patients)	
	*n* *=* *52 patients (%)*
Stroke	35 (67.3)
Myocardial infarction	10 (19.2)
Peripheral embolism	4 (7.7)
Aorta	2 (3.8)
Precerebral thrombi	2 (3.8)
*Stroke (35 patients)*	*n* *=* *41 thrombi (%)*
Complete Occlusion	35 (85.4)
Free-floating	2 (4.9)
Wall-adherent Thrombi	4 (9.75)
*Vessel*	
Internal carotid artery	9 (22)
Middle cerebral artery (MCA-M1)	7 (17)
Middle cerebral artery (MCA-M2)	9 (22)
Middle cerebral artery (MCA-M3)	3 (7.3)
Middle cerebral artery (MCA-M4)	1 (2.4)
Vertebral artery	3 (7.3)
Basilar artery	3 (7.3)
Anterior cerebral artery (ACA-A2)	3 (7.3)
Common carotid artery	2 (4.9)
Posterior cerebral artery (PCA-P3)	1 (2.4)
*Myocardial Infarction (10 patients)*	*n* *=* *10 patients (%)*
Received cardiac catheterization	10 (19.6)
Stenosis	8 (80)
Thrombosis	2 (20)
NSTEMI	8 (80)
STEMI	2 (20)
*Peripheral embolism (4 patients)*	*n* *=* *5 thrombi (%)*
Complete Occlusion	5 (100)
Radial artery	2 (40)
Left princeps pollicis artery	1 (20)
Left common palmar digital artery	1 (20)
Left superficial femoral artery	1 (20)
*Others (4 patients)*	*n* *=* *4 thrombi (%)*
Aorta (free-floating thrombi)	2 (50)
Precerebral arteries	2 (50)
Subclavian artery (free-floating)	1 (50)
Vertebral artery (wall-adherent)	1 (50)

Abbreviations: *SD* standard deviation; *(N) STEMI* (non-) ST-elevation myocardial infarction

### Incidence of COVID-19 Associated ATE

Of the initial 3267 hospitalized COVID-19 patients included in the study, 102 (3.1%) patients presented with 110 ATE. The 102 patients with ATE had 44 cerebral infarctions, 51 myocardial infarctions, four thrombi in precerebral arteries, four occlusions of peripheral arteries, three mesenteric ischemia, two thrombi in the aorta, one splenic infarction, and one kidney infarction.

**Fig. 2:**
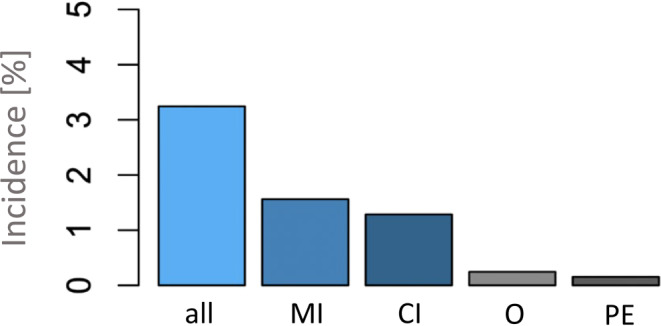
Incidences of ATEs. Incidences of ATE by affected vascular system of all centers. Abbreviations: ATE arterial thrombotic event; MI myocardial infarction; CI cerebral infarction; O other; PE Peripheral embolism

Reported incidences differed mainly between centers with CSCs and centers primarily focused on infectious diseases, but interestingly also considerably in-between centers with CSCs. Mostly affected areas were cardiac and craniocervical circulation. Detailed incidences and affected system are presented in Fig. 2.

### Timeline of ATE

**Fig. 3:**
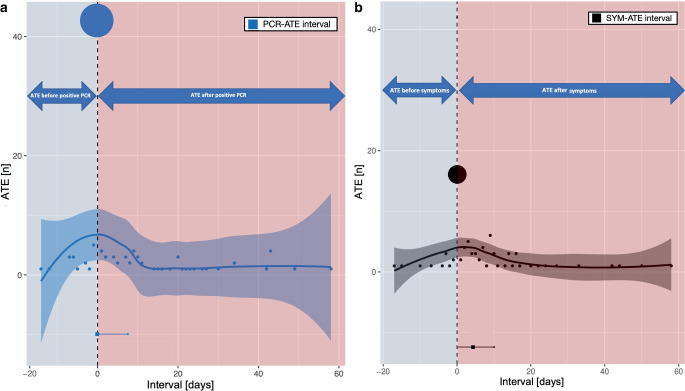
Timeline of ATE in respect to COVID-19 symptoms and positive RT-PCR testing. Time course of ATE occurrence relative to positive PCR-testing for COVID-19 (**a**) and onset of COVID-19 symptoms (**b**). Colored bands indicate the respective 95%-confidence intervals. Growing diameter of the dots indicates amount of ATE per indexed day. The vertical dotted line represents either the time of positive PCR test (**a**) or the time of first symptoms (**b**). Abbreviations: ATE arterial thrombotic event; PCR polymerase chain reaction

ATE occurred at a median of 4 (range, −5–58; interquartile range 0–10) days after the onset of typical respiratory symptoms of COVID-19 and at a median of 0 (range, −7–58; interquartile range 0–8) days after the first positive PCR test result. Negative values in this context indicate that some ATEs occurred prior to the patients’ positive PCR testing or the onset of typical respiratory symptoms. Most ATE occurred at the beginning and the first week of the infection, although events were observed over the whole hospitalization of patients (maximum two months). Visualization of the time course of ATE associated with a COVID-19 infection is shown in Fig. 3.

### Morphology

Detailed imaging data of occurred ATE were available in 52 of 102 ATE cases. In some cases of myocardial infarction (*n* = 20/19.6%), ATE was diagnosed primarily based on clinical criteria in addition to biomarker elevations or electrocardiographic changes. DSA was only performed if the endovascular intervention was necessary. Performance and availability of detailed imaging data differed between participating centers due to different clinical specialization profiles.

Overall, the 52 in detail analyzed cases showed a total of 60 thrombi: 41 in ischemic strokes, 10 myocardial infarctions, four occlusions of peripheral arteries, two thrombi in precerebral arteries, and two thrombi in the aorta. Four of these patients had free-floating thrombi in the carotid arteries and aorta, which refers to a thrombus that is suspended within the lumen of the artery unattached or only minimally attached to the vessel wall at one end [15]. One patient had a free-floating thrombus in the subclavian artery found incidentally. Only ten patients with acute myocardial infarction received catheterization, showing two complete thrombosis and eight highly stenotic vessels with one free-floating, partially obstructing thrombosis.

**Fig. 4:**
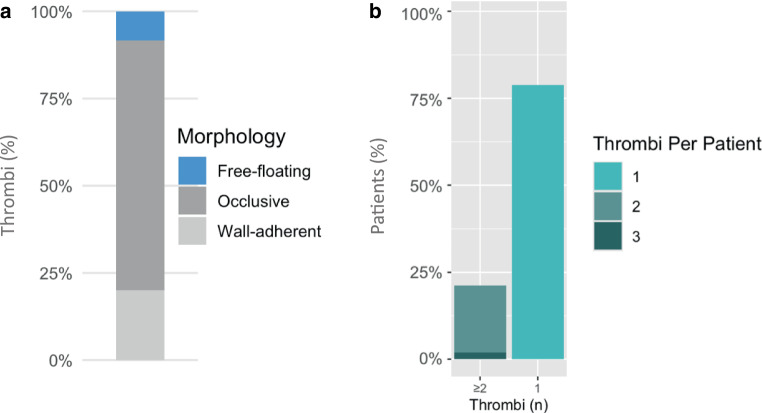
Morphology of ATE. **a** shows the morphological characteristics of all analyzed thrombi and **b** shows the percentage of patients with multiple thrombi

To get an impression of the occurrence rate of atypical occlusion characteristics, we generated an additional analysis including only the evaluated, i.e. imaged clots. As shown in Fig. 4 the overall percentage of free-floating appearance was 10.0%, and the occurrence of multiple emboli was 21.2%, to all evaluated clots, respectively.

**Fig. 5:**
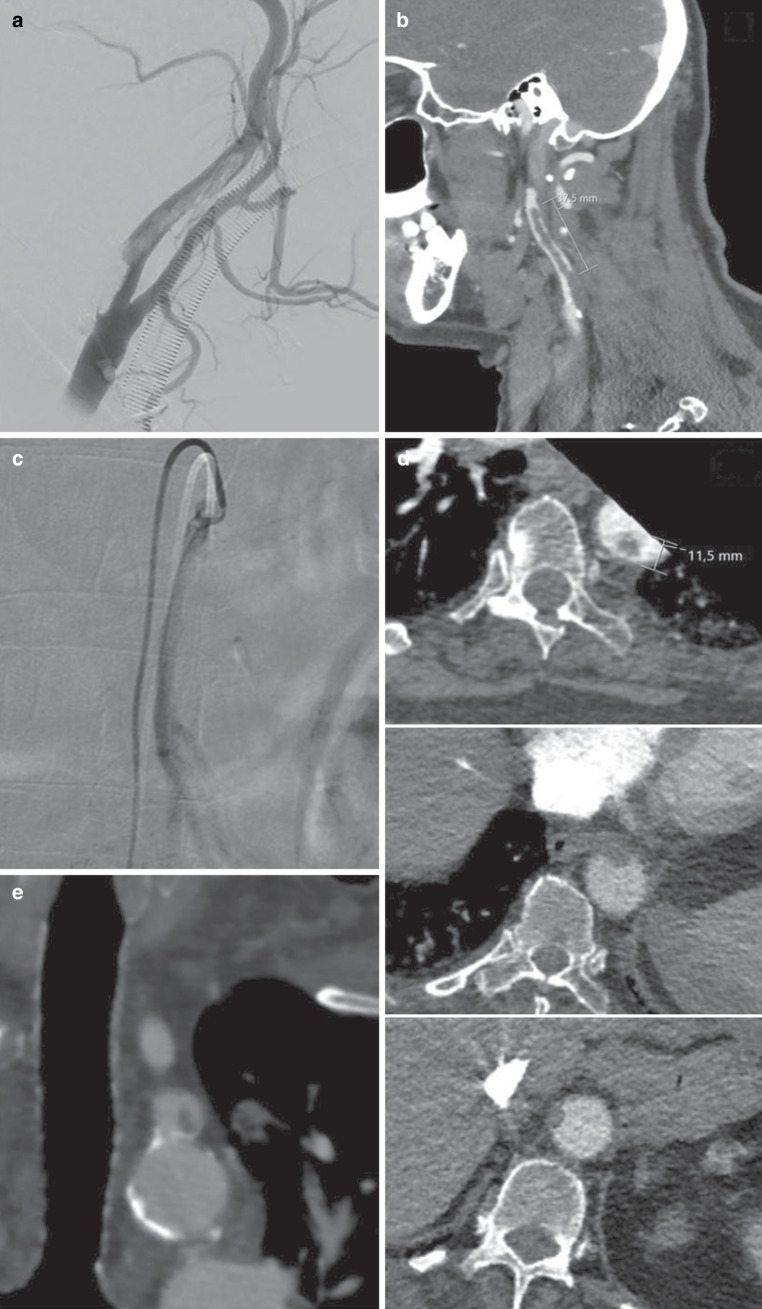
Imaging morphology of ATE co-occurring with COVID-19 infection. **a** Digital subtraction angiography (DSA) shows a massive thrombus of the internal carotid artery, that is partially wall-adherent to a non-ulcerating plaque and with a long free-floating portion inside the internal carotid artery. **b** CT-angiography (CTA) of the internal carotid artery in the same patient shows the ≈ 3.7 cm long floating portion of the thrombus. **c** DSA shows another example of a partially wall-adherent and floating thrombus without visible atherosclerotic changes of the superior mesenteric artery. **d** CTA of the aorta shows multiple wall-adherent thrombi. **e** CTA shows partially wall-adherent and floating thrombi of the left subclavian artery

Figure 5 shows some examples of the typical appearance of these large, partially free-floating wall-adherent non-fully-occlusive thrombi in different body regions.

## Discussion

In this multicenter study, we analyzed the occurrence and characteristics of ATE in 3267 hospitalized COVID-19 patients across five tertiary care centers. Our key findings are:

1. The overall ATE incidence across all body regions was 3.1% in the studied cohort. However, incidences varied depending on the affected arterial vascular system and the specification of the admitting center (e.g., infectious diseases or comprehensive stroke centers).
2. The peak of ATE occurrence was observed on day four after first symptoms and on the day of positive PCR testing, hinting that the first week of infection is the most vulnerable period for ATE development.
3. Unusual clot features like prominent free-floating appearance or multiple vessel occlusions are much more frequent than in other causes of embolic events.

Similarly to previous evidence, the co-occurrence of ATE among hospitalized patients with COVID-19 (3.1%) was notably higher than in viral respiratory infections like influenza (0.2%) or SARS-CoV‑1 (0.75%) [17, 18]. Our reported ATE incidence aligns with the majority of previous studies, especially of a recent meta-analysis with an event rate of 3.9% [19] or a nationwide cohort study with a reported event rate of about 2% [6]. It also confirms the dominance of cerebro- and cardiovascular events, although peripheral events also occur. In addition to previous reports, we showed that the incidence of ATE varied based on the specialty of the hospital to which patients were admitted. Even between centers with similar specialty characteristics, e.g., hospitals with CSCs, the incidence varied between 3 and more than 5%. However, the average incidence of ATE of the participating hospitals with different specialties in this study, covering almost all hospitalized COVID-19 patients of a large metropolitan area, should robustly reflect the real occurrence rate of ATE in this patient group.

In accordance with previous reports, most ATE occurred in patients who were already COVID-19 positive and symptomatic, although there were several patients whose ATE preceded a positive COVID-19 test or related respiratory symptoms.

The highest incidence was observed during the first week of infection, about four days after the first symptoms and on the day of the first positive PCR test. This is in line with reports of a more than 20-fold increased risk of ATE in the first week of infection and the higher than usual risk in the following weeks after the infection [6, 20]. The first week after onset of COVID-19 symptoms likely is the most important for ATE development as previous evidence suggests a surge in inflammatory cytokines [21], platelet activation [22], and endothelial injury [23] during this early phase of infection.

While most ATE presented typical embolic obstruction of peripheral arteries, some cases showed unusual distinctive clot features previously reported in the literature. Of these, the most noticeable features are large thrombi with intraluminal free-floating portions or wall-adherent thrombi in large vessels like the aorta and carotids [9–15]. Although it is difficult to prove a direct pathophysiological association with COVID-19 infection, the high occurrence of such features in COVID-19 patients suggests a possible association with the infection’s pathophysiology. Usually, these free-floating thrombi are observed rarely in about 1–2% and typically occur on ulcerated or stenosing plaques [15, 24–26]. In a comprehensive literature review, nearly half of the patients with free-floating thrombi were found to be in a hypercoagulable state, frequently associated with malignancy [25]. However, the underlying pathology of free-floating thrombi often remains unclear, and no data is available on their incidence in diseases like COVID-19, such as Influenza. In our study, the occurrence rate of free-floating clots was increased (10.0%), as was the occurrence of multiple thrombi (21.2%), which is assumed to be lower at about 15–20% according to data from, for example, the German Stroke Registry [27]. So overall, our data seems to be in line with previous analyses of thrombi adherent to vessel walls, which showed intima inflammatory infiltrates, i.e., vascular lesions, so that causally, a combination of endotheliitis and a hypercoagulation state in COVID-19 can be presumed [28–30] and might be additionally supported by our results. This hypothesis is further strengthened by recent findings of SARS-CoV‑2 in vascular early-stage lesions [30].

### Strengths and Limitations

The main strength of our study is the large number of patients, representing the vast majority of hospitalized COVID-19 patients in a metropolitan area. ATE in all body regions were recorded and the available image data was evaluated in detail. However, the retrospective design has limitations, such as incomplete or limited data quality. Additionally, the ATE rate in this study may represent a conservative estimate. Embolic events may be underreported in unconscious or intubated patients. Furthermore, the chosen rather conservative inclusion period of seven days prior to confirmed COVID-19 diagnosis, while helpful in ensuring a temporal association between ATEs and COVID-19 hypercoagulability, may exclude a subset of COVID-19-related ATEs. Thus, the true incidence of ATEs hospitalized patients with COVID-19 could be higher than reported in this study. Limited imaging data also allowed only descriptive and cautious conclusions about distinctive clot features.

## Conclusion

This extensive retrospective, multi-center analysis of hospitalized COVID-19 patients with ATE confirms the systemic nature of COVID-19, with ATE occurring in all vascular regions, particularly affecting the heart and brain. COVID-19 associated ATE possibly inherit distinct imaging features, such as bulky free-floating clot masses and multiple occlusions, which, however, requires more evidence. ATE occur most frequently during the first week around the COVID-19 diagnosis.

## Supplementary Information

Supplementary demographical and clinical data of the study cohort.

## Abbreviations

ATE: arterial thrombotic event
CSC: comprehensive stroke centers
CID: centers of infectious diseases
