# Supplementary material for: A Retrospective Multicenter Study of Arterial Thromboembolic Events in Hospitalized COVID-19 Patients: Incidence and Imaging Characteristics
Source: Clin Neuroradiol. 2025 Mar 4;35(3):511–9. doi: 10.1007/s00062-025-01503-w (PMC12454460; doi:10.1007/s00062-025-01503-w)
Supplement: Supplementary file 1 — Supplementary demographical and clinical data of the study cohort. [file 62_2025_1503_MOESM1_ESM.docx]

**Supplementary Material for:**

**A retrospective multicenter study of arterial thromboembolic events in COVID-19 patients: Incidence and imaging characteristics**

By *blinded*for*review* et al.

**Supplement Material**

**Participants**

Group demographics and clinical characteristics for the whole cohort and split for the specialization of the centers, i.e., infectious diseases and comprehensive stroke unit, are presented in Supplementary Table 1.

**Supplementary Table 1. Demographical and clinical data of the study cohort.**

|  | **All COVID-19+**  **ATE+**  ***n=102 (%)*** | **CSC COVID-19+ ATE+**  ***n=84 (%)*** | **CID COVID-19+ ATE+**  ***n=18 (%)*** |
| --- | --- | --- | --- |
|  |  |  |  |
| Age (mean ± SD) | 72.01 ± 15.64 | 72.85 ± 20.98 | 68.11 ± 15.10 |
| Sex (f/m) | 39/63 | 33/51 | 6/12 |
| SARS-Cov-2 confirmation by test | 102 (100) | 84 (100) | 18 (100) |
|  |  |  |  |
| **Cardiovascular risk factors** | 85 (83.3) | 72 (55.7) | 13 (72.2) |
| Arterial hypertension | 57 (55.9) | 51 (60.7) | 6 (33.3) |
| Diabetes mellitus | 65 (63.7) | 52 (61.9) | 13 (72.2) |
| Dyslipidemia | 11 (10.8) | 6 (7.1) | 5 (27.8) |
| Smoking | 11 (10.8) | 8 (9.5) | 3 (16.7) |
| Hypercholesterolemia | 25 (24.5) | 17 (20.2) | 8 (44.4) |
| Adiposities | 15 (14.7) | 13 (15.5) | 2 (11.1) |
| Atrial fibrillation | 19 (18.6) | 16 (19) | 3 (16.7) |
| CHD | 29 (28.4) | 24 (28.6) | 5 (27.8) |
| **Treatment at admission** |  |  |  |
| none | 42 (41.2) | 35 (41.7) | 7 (38.9) |
| Antiplatelet | 42 (41.2) | 36 (42.9) | 6 (33.3) |
| ASA 100mg | 34 (33.3) | 28 (33.3) | 6 (33.3) |
| Clopidogrel 75mg | 8 (7.8) | 8 (9.5) | 0 (0) |
| Prasugrel | 0 (0) | 0 (0) | 0 (0) |
| Ticagrelor | 0 (0) | 0 (0) | 0 (0) |
| Oral anticoagulation | 14 (13.7) | 11 (13.1) | 3 (16.7) |
| Apixaban |  |  |  |
| 2.5 mg | 5 (4.9) | 4 (4.8) | 1 (5.6) |
| 5 mg | 4 (3.9) | 3 (3.6) | 1 (5.6) |
| Dabigatran |  |  |  |
| 110 mg | 1 (1) | 1 (1.2) | 0 (0) |
| Rivaroxaban |  |  |  |
| 15 mg | 2 (2) | 1 (1.2) | 1 (5.6) |
| 20 mg | 2 (2) | 2 (2.4) | 0 (0) |
| Vitamin K antagonists | 4 (3.9) | 4 (4.8) | 0 (0) |
| Heparin | 4 (3.9) | 4 (4.8) | 0 (0) |
| Enoxaparin 2.000 IE | 1 (1) | 1 (1.2) | 0 (0) |
| Enoxaparin 4.000 IE | 2 (2) | 2 (2.4) | 0 (0) |
| Tinzaparin 14.000 IE | 1 (1) | 1 (1.2) | 0 (0) |
| Statin | 56 (54.9) | 51 (60.7) | 5 (27.8) |
| Atorvastatin |  |  |  |
| 10 mg | 1 (1) | 1 (1.2) | 0 (0) |
| 10 mg | 1 (1) | 1 (1.2) | 0 (0) |
| 20 mg | 5 (4.) | 4 (4.8) | 1 (5.6) |
| 40 mg | 25 (24.5) | 22 (26.2) | 3 (16.7) |
| 80 mg | 6 (5.9) | 6 (7.1) | 0 (0) |
| Simvastatin |  |  |  |
| 20 mg | 4 (3.9) | 4 (4.8) | 0 (0) |
| 40 mg | 11 (10.8) | 10 (11.9) | 1 (5.6) |
| 80 mg | 3 (2.9) | 3 (3.6) | 0 (0) |
| Rosuvastatin |  |  |  |
| 10 mg | 1 (1) | 1 (1.2) | 0 (0) |
|  |  |  |  |
| **Prothrombotic risk factors** |  |  |  |
| Active cancer | 9 (7.8) | 7 (8.3) | 2 (11.1) |
| Sepsis | 2 (2) | 0 (0) | 1 (5.6) |
| Chronic renal failure | 15 (14.7) | 13 (15.5) | 2 (11.1) |
| DIC | 0 (0) | 0 (0) | 0 (0) |
| MV prior to ATE | 9 (8.8) | 7 (8.3) | 2 (11.1) |
| Duration of MV prior to  ATE (mean ± SD) *n=9* | 13.01 ± 14.95 | 5.73± 4.8 | 38.5 ± 9.5 |
| Prothrombotic drugs prior  to ATE | 18 (14.7) | 14 (16.7) | 4 (22.2) |
| ICU duration prior ATE  (mean ± SD) *n=12* | 12.39 ± 15.7 | 6.77 ± 7.82 | 40.5 ± 16.26 |
|  |  |  |  |
| **COVID-19 severity** |  |  |  |
| Respiratory symptoms | 81 (79.4) | 67 (79.8) | 14 (77.8) |
| Radiological lung  manifestations | 83 (81.4) | 68 (81) | 15 (17.9) |
| In-hospital mortality | 35 (34.3) | 27 (32.1) | 8 (44.4) |

Abbreviation: ASA, acetylsalicylic acid; ATE, arterial thromboembolic event; CID, center of infectious diseases; CSC, comprehensive stroke centers; DIC, disseminated intravascular coagulation; f, female; m, male; mg, milligram; MV, mechanical ventilation; SD, standard deviation; CHD, chronic heart disease.
